# Obesity-Induced Heart Rate Variability Impairment and Decreased Systolic Function in Obese Male Dogs

**DOI:** 10.3390/ani10081383

**Published:** 2020-08-10

**Authors:** Wanpitak Pongkan, Wannida Jitnapakarn, Warunee Phetnoi, Veerasak Punyapornwithaya, Chavalit Boonyapakorn

**Affiliations:** 1 Department of Veterinary Biosciences and Veterinary Public Health, Faculty of Veterinary Medicine, Chiang Mai University, Chiang Mai 50100, Thailand; p.wanpitak@gmail.com (W.P.); wannida.jpk@yahoo.com (W.J.); kiriten10@gmail.com (W.P.); 2 Integrative Research Center for Veterinary Circulatory Sciences, Faculty of Veterinary Medicine, Chiang Mai University, Chiang Mai 50100, Thailand; 3 Veterinary Cardiopulmonary Clinic, Small Animal Hospital, Faculty of Veterinary Medicine, Chiang Mai University, Chiang Mai 50100, Thailand; 4 Department of Food Animal Clinic, Faculty of Veterinary Medicine, Chiang Mai University, Chiang Mai 50100, Thailand; pveerasak.r@gmail.com; 5 Department of Companion Animal and Wildlife Clinic, Faculty of Veterinary Medicine, Chiang Mai University, Chiang Mai 50100, Thailand

**Keywords:** obesity, oxidative stress, cardiac function, heart rate variability, dog

## Abstract

**Simple Summary:**

Obesity in dogs can induce many adverse health effects including musculoskeletal problems, respiratory distress, metabolic syndrome, and cardiovascular diseases. In humans with obesity, heart rate variability (HRV) is used to identify and predict the risk of cardiovascular diseases. However, this predictive tool has never been used in veterinary medicine, and the relationship between obesity and HRV has rarely been investigated. In this study, we investigated HRV, plasma oxidative stress (MDA), and cardiac function in obese male dogs. We hypothesized that obese male dogs have decreased cardiac function and impaired HRV compared to non-obese dogs. Our study found that obese dogs have decreased cardiac systolic function and impaired HRV, as indicated by reduced percentages of cardiac contraction and impaired cardiac autonomic activity compared to non-obese dogs. We concluded that obesity can decrease systolic function and cause HRV impairment, which might increase the risk of cardiovascular disease in dogs. In addition, HRV might be used as a predictive or prognostic tool in the prevention of cardiovascular disease in obese dogs.

**Abstract:**

Obesity can induce cardiovascular diseases in both humans and animals. Heart rate variability (HRV) is an indicator of sympathovagal balance and is used to identify cardiovascular diseases in humans. However, HRV and cardiac function have rarely been investigated in obese dogs. This study investigated the effect of obesity on oxidative stress, HRV, and cardiac function in obese and non-obese dogs. The nine-scale body condition score (BCS) system was used to determine obesity. Thirty small breed dogs were divided into a normal weight group (*n* = 15) and an obese group (*n* = 15). All dogs underwent physical examination, plasma malondialdehyde (MDA) measurement, electrocardiography, echocardiography, and two hours of Holter monitoring. This study found that obese dogs had increased plasma MDA and sympathovagal imbalance, which was indicated by impaired time and frequency domains compared to normal weight dogs. Although cardiac function was within normal limits, the echocardiographic study found that the obese dogs had reduced cardiac wall thickness and lower systolic function, as indicated by a reduction in %ejection fraction, %fractional shortening, increased left ventricular (LV) internal diameter during systole, and LV end-systolic volume compared to normal weight dogs. This study concluded that obesity in dogs can induce increased plasma oxidative stress, impaired HRV, and reduced cardiac systolic function compared to non-obese dogs.

## 1. Introduction

Obesity is a nutritional disorder that can be found in dog populations [1]. In one published study, 29–34% of dogs were classed as overweight and 5–8% were judged obese [2]. A 25–52% prevalence of obese dogs has been reported in developed countries [3]. Another study suggested that there are various methods for assessing body composition, observed body weight (BW) to standard body weight and determining BCS category [4]. Regarding comparison of observed BW to standard BW, if the observed body weight is greater than 15% of the standard BW, the dog is considered obese [5]. BCS category is the most widely accepted clinical method which uses visual and palpable characteristics [6]. Several previous studies have demonstrated that obesity in dogs and cats is related to metabolic impairment, musculoskeletal problems, respiratory distress, and cardiovascular disease [1,7,8,9,10]. A previous echocardiographic study in dogs demonstrated that obesity can induce left ventricular hypertrophy [11]. Moreover, the effect of obesity on the heart in a rat model demonstrated that obese-insulin resistance can lead to LV contractile dysfunction, increased oxidative stress, cardiac mitochondrial dysfunction and cardiac sympathovagal imbalance or HRV impairment [12].

HRV is a non-invasive assessment used to evaluate health status and neurocardiac function. Measurements include both a time domain and a frequency domain, where the time domain consists of standard deviation of all NN intervals (SDNN), Standard deviation of all R-R intervals (SDRR), Standard deviation of the averages of NN intervals in 5-min (SDANN), Percentage of successive NN intervals >50 msec (pNN50), and Root mean square of the sum of the squares of differences between adjacent NN intervals (RMSSD), while the frequency domain consists of total power (TP), ultra-low frequency (ULF), very low frequency (VLF), low frequency (LF), high frequency (HF), low frequency in normalized unit (LFnu), high frequency in normalized unit (HFnu), and low frequency per high frequency (LF/HF) [13,14]. HRV impairment has been used as an index of cardiac sympathovagal balance [15] and of non-cardiovascular diseases in humans, e.g., liver diseases and kidney diseases in obese children [15,16,17,18].

In veterinary medicine, HRV has been found to vary by species, age, breed, and emotion [14,19,20,21,22]. Both a time domain and a frequency domain have been used in animal models [14,19,20,21,23]. Previous studies have demonstrated that dogs, calves, and humans have similar LF, HF, and LF/HF ratios, whereas these values are completely different in rabbits and rats [23]. Additionally, a previous study using time-domain HRV demonstrated that facial conformation in dogs can affect the vasovagal tonus index (VVTI)and that congestive heart failure can reduce VVTI [20]. However, a study in two different breeds of healthy dogs demonstrated that HRV (frequency-domain) parameters were not different among strains based on sex, age, and body weight [19]. Other studies of dogs with myxomatous mitral valve degeneration (MMVD) and heart failure have reported that these dogs demonstrated HRV impairment, as indicated by the decreased RMSSD, SDNN, MeanNN, pNN50, and VVTI indices and increased LFnu and LF/HF ratios [24,25,26,27]. Additionally, a study in rats reported that an increased LF/HF ratio is an indicator of cardiac sympathovagal imbalance [12]; the LF/HF ratio has also been shown to be increased in overweight humans [28]. Similarly, a previous study found a relationship between HRV impairment and increased oxidative stress [18].

In addition, a previous study demonstrated that obesity can induce an increase in reactive oxygen species (ROS) production as superoxide, hydroxyl radical, and hydrogen peroxide derived from the increase in mitochondrial respiration [29]. Moreover, excessive fat can induce accumulation of cellular damage, which generates ROS in tissues and increases the lipid peroxidation rate. These changes generate an end product known as malondialdehyde (MDA) [29,30]. Previous studies have demonstrated that increasing oxidative stress clearly shows a contribution to the pathology of cardiovascular disease [31] and HRV impairment [30]. Experimental studies in animal models indicate that ROS can affect HRV and other cardiovascular endpoints [32,33]. Moreover, studies in rat models have shown that obesity-related insulin resistance is a risk factor for developing cardiac sympathovagal dysregulation, as indicated by an increase in the LF/HF ratio [34], whereas MDA is significantly correlated with the LF/HF ratio in humans with diabetes [35]. Studies have also shown that obesity causes an increase in oxidative stress and insulin resistance which strongly affects cardiac autonomic regulation, causes cardiac systolic dysfunction, and induces cardiovascular diseases [32,33,35,36]. Thus, whether the observations are made in humans or in animals, evaluation of HRV and oxidative stress can provide information about cardiac function and can help predict diseases that can occur in obese dogs.

There have been many studies about the effects of obesity on metabolic parameters, cardiac function, HRV and oxidative stress in mice, rats and humans [34,37,38,39], but these parameters have rarely been explored in dogs. In the present study, we investigated the effects of obesity on blood profile, cardiac performance, as indicated by echocardiography and electrocardiography, HRV, and oxidative stress in obese intact male dogs compared with non-obese intact male dogs. We hypothesized that obese intact male dogs have decreased cardiac performance, increased oxidative stress, impaired HRV, and altered blood profiles compared to non-obese intact male dogs.

## 2. Materials and Methods

### 2.1. Animal Model and Research Protocol

In this study, clinically healthy dogs who visited a small animal hospital were given general screening tests including history taking, physical examination, cardiovascular disease evaluation using echocardiography and electrocardiography, thoracic radiography, and blood collection. Dogs with one or more asymptomatic and/or symptomatic systemic diseases, metabolic diseases, cardiovascular diseases (e.g., systemic hypertension, congenital or acquired cardiac diseases, and abnormal ECG), chronic inflammatory diseases (e.g., orthopedic diseases), respiratory disorders, endocrine disorders, chronic kidney disease, cryptorchidism or tumors were excluded from this stu. In this clinical study, thirty healthy dogs from two small breeds (Pomeranian and Chihuahua) aged between 1.8 and 8.0 years were divided into two groups: normal weight and obese. The first group of 15 (9 Chihuahuas and 6 Pomeranians) were normal weight, i.e., not obese intact male dogs, whereas the second group of 15 (8 Chihuahuas and 7 Pomeranians) were obese intact male dogs. The body condition of all the dogs was determined using the nine-scale body condition score (BCS) system [40]. Scores was allocated by the same principle investigator. Dogs with a BCS of 7–9 were considered to be obese, whereas those with a BCS of 4–5 were considered to be normal weight dogs. Borderline obese dogs, i.e., dogs in the gray zone (BCS 6), were excluded from this study. All the dogs underwent a physical examination including blood collection, electrocardiography, echocardiography, a Holter recording for 2 h to investigate cardiac function and cardiac sympathovagal balance, and plasma oxidative stress measurement. This study was performed at the Small Animal Hospital, Faculty of Veterinary Medicine, Chiang Mai University, Thailand. All owners signed an informed consent form and all experiments was approved by the Ethics Committee of the Laboratory Animal Center, Faculty of Veterinary Medicine, Chiang Mai University, Thailand (Ethical number: S24/2561).

### 2.2. Physical Examination

All dogs were evaluated for common health status by physical examination (inspection, palpation, percussion, and auscultation) including general appearance and vital signs.

### 2.3. Blood Pressure Evaluation

Blood pressure (BP) measurements were made using an oscillometer (CARESCAPE^TM^ V100, GE Healthcare, Milwaukee, WI, USA) and evaluated following American College of Veterinary Internal Medicine (ACVIM) consensus statement guidelines for the identification, evaluation, and management of systemic hypertension in dogs and cats [41]. Either the left or the right forelimb circumference was measured using a cuff corresponding to 40% of the circumference of the limb. Systolic and diastolic blood pressure were recorded. Dogs with systemic hypertension were excluded from this study. The average of four consecutive BP measurements was used for statistical analysis.

### 2.4. Electrocardiographic Evaluation

In this study, electrocardiographic examination was conducted using six standard limb leads (MAC^TM^ 800, GE Healthcare, Milwaukee, WI, USA) and was performed in a quiet room. Unsedated dogs were allowed to stabilize for 15 min prior to the ECG. ECG alligator clips were attached to the skin of the dog’s limbs (elbow position at forelimbs and hock position at hindlimbs) following color coding from the ECG machine. Alcohol was applied to the attachment site to better transmit the electrical activity of the heart to the electrode. A paper speed of 25 mm/s and a calibration of 10 mm/1 mV were used in this study. In addition, lead II was used to measure heart rate, cardiac rhythm, mean electrical axis (MEA), amplitude (mV) and duration (ms) of P wave and QRS complex, PR interval (ms), QT interval (ms) and ST segment amplitude (mV).

### 2.5. Cardiac Function Determination

Cardiac walls, cardiac chambers, and cardiac function were evaluated using echocardiography (CX 50 xMatrix, Philips^TM^, Bothell, WA, USA) in 2D and 2D-guided M-mode technique. The thoracic wall was shaved after which the dog was allowed to stabilize for one to two minutes. Echocardiographic examination of the right parasternal short axis (at the base of the heart and at the level of the papillary muscles), and right parasternal long axis was conducted by an experienced veterinarian with randomized and blinded techniques. Cardiac wall thickness and left ventricular (LV) dimensions were measured, including the left ventricular posterior wall (LVPW), the interventricular septum (IVS), the left ventricular internal diameter (LVID) in both systole and diastole, the width of the left atrium (LA) and the size of the aorta (AO), which gave the LA/AO ratio. Finally, the %fractional shortening (%FS) and the %ejection fraction (%EF) were calculated. All M-mode average cardiac chambers were normalized by body weight using the Cornell allometric scale for dogs [42].

### 2.6. Blood Profiles and Oxidative Stress Determination

Blood was collected by venipuncture and divided into three fractions. The first fraction was placed in tubes containing potassium ethylene diamine tetra-acetic acid (EDTA) for complete blood count (CBC) analysis. The second fraction was placed in tubes with silica dioxide (a clot activator) for blood chemistry analysis, and the third was collected as plasma and stored at −80 °C for malondialdehyde (MDA) analysis using an MDA assay kit (Thiobarbituric acid colorimetric method), (Elabscience^®^, Houston, TX, USA).

### 2.7. Heart Rate Variability (HRV) Determination

The dogs were placed in a quiet separate room where they were connected to a Holter ECG recorder (SEER^TM^ 1000, GE Healthcare, Milwaukee, WI, USA) for 150 min using standard chest leads. The HRV data were collected in the morning (8.00–12.00 AM). After completing the recording, the ECG waveform was automatically evaluated by the MAR^TM^ program (GE Healthcare, Milwaukee, WI, USA), after which the ECG artifacts and premature complexes were manually removed. Then a 120 min continuous ECG segment was selected and analyzed using the MAR^TM^ program. Power spectra of RR interval variability were obtained using the Fast Fourier Transform (FFT) algorithm, and the three major oscillatory components including high frequency (HF) (0.15–0.4 Hz), low frequency (LF) (0.04–0.15 Hz) and very low frequency (VLF) (0.003–0.04 Hz) were determined. Parasympathetic activity was regarded as HF, whereas associations of sympathetic and parasympathetic activity were regarded as LF [43,44]. In order to minimize the effect of changes in total power on the LF and HF components, LF and HF were expressed as normalized units (LFnu and HFnu) by dividing either LF or HF by the total power minus VLF. The LF/HF ratio was considered an index of cardiac sympathovagal tone balance [15,45]. An increased LF/HF ratio indicates a cardiac sympathovagal imbalance (increased sympathetic tone relative to parasympathetic tone). The time domain (Mean NN, SDNN, SDANN, ASDNN, RMSSD, and pNN50) and the frequency domain (VLF, LF, HF, and LF/HF) HRV were analyzed using the MAR^TM^ program.

### 2.8. Statistical Analysis

All data are presented as the mean ± SE (median, minimum-maximum). All representative values in obese dogs (*n* = 15) were compared to those in non-obese dogs (*n* = 15). The Student’s t test was used to determine differences in means between the obese and non-obese groups. All statistical analyses were carried out using R statistical software version 3.6.3 (R core team, 2020). Assumptions for the Student’s t test, including normality and homogeneity of variances, were tested using the Shapiro–Wilk test and Levene’s test, respectively. Statistical significance was established as *p*-values less than 0.05 (*p* < 0.05).

## 3. Results

### 3.1. Body Weight and Body Condition Scores of Obese Dogs Were Higher than Non-Obese Dogs

There were no statistically significant differences in physical examination parameters (age, systolic blood pressure, diastolic blood pressure, mean arterial blood pressure, heart rate, pulse rate, respiratory rate, and rectal temperature) between the normal weight and obese groups. However, the body weight and the body condition score were higher in the obese group when compared to the normal weight group (Table 1).

### 3.2. Obese Dogs Produced More Oxidative Stress (MDA) than Non-Obese Dogs

There was no difference in blood profile parameters between the groups with the exception of PCV, RBC, and albumin, which were higher in the obese group compared to the normal weight group. However, PCV and RBC were still in the normal range, but hyperalbuminemia was found in the obese group, which could have been due to dehydration or high protein consumption among other factors. In addition, plasma oxidative stress indicated by malondialdehyde (MDA) was higher in the obese group compared to the non-obese group (Table 2).

### 3.3. Obese Dogs Have Reduced Cardiac Systolic Performance Compared to Non-Obese Dogs

Cardiac function and cardiac dimensions were evaluated in this study. Between obese and normal weight dogs, a statistically significant difference in the six values was observed: %EF, %FS, normalized LVIDs as well as normalized LVIDd, EDV, and ESV. In this study, dogs in the obese group demonstrated reduced cardiac systolic performance, as indicated by statistically significantly lower echocardiographic %EF and %FS when compared to the non-obese group. Moreover, the obese group had statistically significantly decreased normalized LVIDs and increased normalized LVIDd, EDV, and ESV when compared to the non-obese group. Other echocardiographic parameters were not statistically significantly different between the groups (Table 3 and Table 4). M-mode echocardiographic parameters were not different between the two breeds within each group (the normal weight and the obese groups) (Table 3 and Table 4). However, %EF, %FS, cardiac chamber (LVIDs and LVIDd) and cardiac volume (EDV and ESV) were statistically significantly different between obese and non-obese dogs of the same breed (Table 5 and Table 6).

### 3.4. Obese Dogs Demonstrated Eccentric Hypertrophy of Left Ventricle Compared to Non-Obese Dogs

Cardiac wall thickness was evaluated using echocardiography. This study found that the normalized values of the left ventricular posterior wall end-systole (LVPWs) and end-diastole (LVPWd) were decreased, whereas LV mass and LV mass index were increased in obese dogs compared to normal weight dogs (Table 3 and Table 4). Cardiac wall thickness from M-mode echocardiographic parameters were not different among breeds within each group. However, cardiac wall (LVPWd and LVPWs) and cardiac mass (LV mass and LV mass index) were statistically significantly different between groups for dogs of the same breed (Table 5 and Table 6).

### 3.5. Obese Dogs Demonstrated Alteration of Cardiac Sympathovagal Imbalance Compared to Non-Obese Dogs

In the time domain, the heart rate variability demonstrated that mean NN, SDNN, SDANN, ASDNN, and RMSSD were statistically significantly lower in the obese group compared to the non-obese group (Table 7). In addition, in the frequency domain, the VLF, LF, and HF were not statistically significantly different between the groups, whereas the LF/HF ratio was statistically significantly higher in the obese group compared to the non-obese group which may indicate impaired HRV or cardiac sympathovagal imbalance in obese dogs compared to the non-obese dog (Table 8).

### 3.6. No Relevant Differences were Found in ECG Morphology between Obese and Non-Obese Dogs

In this study, we found that ECG morphology, cardiac rhythm and MEA in obese and non-obese dogs were not statistically significantly different in the two groups. In addition, we found that the R-wave amplitude in the obese group was statistically significantly higher compared to the normal weight group. However, all ECG parameters in both obese and non-obese dogs were within normal limits (Table 9).

## 4. Discussion

This study explored and evaluated HRV, cardiac function, and plasma MDA in normal weight dogs (defined as BCS 4–5) compared to obese dogs (defined as BCS 7–9). This study focused on small breeds of dog, namely Pomeranian and Chihuahua, and included similar numbers of each breed. Dogs were divided into two groups based on obesity status. The two major findings of this study are as follows. First, obese dogs have increased plasma oxidative stress, MDA, when compared to non-obese dogs. Second, obese dogs demonstrate reduced cardiac systolic performance, increased cardiac sympathovagal imbalance, and increased cardiac wall thickness compared to non-obese dogs.

Our study investigated the effect of obesity on HRV, cardiac function, and plasma oxidative stress in obese dogs compared to normal weight dogs. Previous studies have reported that obesity impacts the cardiovascular system [1,12,18] and causes oxidative stress [34]. Obesity is a major contributor to several metabolic disturbances related to oxidative balance [48]. In addition, obesity increases myocardial metabolism and the lipid peroxidation rate [29]. The present study found that plasma MDA level, a secondary product of lipid peroxidation, was significantly increased in the obese group. MDA is a product of the lipid oxidation process, which is elevated in many conditions such as dysfunction of mitochondria and cardiomyocyte dysfunction. Although there is no generally accepted normal range of MDA in healthy dogs, the doubling of MDA levels in obese compared to non-obese dogs might be the result of a lipid peroxidation and oxidative imbalance in the obese animals. In addition, previous studies of ten healthy dogs of various large breeds reported a range of plasma MDA values between 1.0 and 1.6 µmol/L using the HPLC system [49,50], whereas another study of six healthy dogs reported values between 1.9 and 2.7 nmol/mL using a TBAR assay kit [51]. Interestingly, in the present study, we found that the range of plasma MDA in fifteen healthy small breed non-obese dogs was between 73 and 81 nmol/mL using the HPLC system, values higher than those in the other studies. That difference might be due to the specific experimental model, the animal breed, and/or differences in assay protocol. Another study reported that an increase in lipid peroxidation has profound consequences in terms of dysfunction of mitochondria, cardiomyocytes, and the heart [52]. As cardiac mitochondria supply energy to produce the proper cardiac contraction and relaxation on a beat to beat basis [34], our study suggests that increasing oxidative stress can negatively affect cardiac function by decreasing %FS and %EF.

Regarding the results of cardiac function and cardiac free wall evaluation using echocardiography, all normalization M-mode echocardiographic parameters in both groups were in the normal range (95% prediction interval). Dogs with cardiovascular diseases which could potentially affect either cardiac function or echocardiographic parameters were excluded from this study. Although no obvious cases of cardiac dysfunction were observed, we did find that the obese group demonstrated decreased cardiac systolic function when compared to the normal weight group. We also found significantly decreased %EF and %FS, increased left ventricular internal diameter during systole (LVIDs) and diastole (LVIDd), EDV, and ESV in the obese group when compared to the normal weight group. The same result was found in comparisons between the normal weight and the obese group for each breed. However, in this study, LVIDs and LVIDd in the normal weight group were slightly below the normal ranges. This difference might have been due to variations between the breed of dogs and/or the small sample size.

Even though the obese dogs did not exhibit obvious cardiac dysfunction, the echocardiographic parameters demonstrate that the hearts of the obese dogs had reduced left ventricular systolic function, as indicated by decreased %fractional shortening, increased LV systolic dimension, and increased LV systolic volume when compared to the normal weight dogs. Moreover, although the obese dogs might have been somewhat dehydrated when their blood profiles were evaluated, a high LV preload was observed in the hearts of the obese dogs, as indicated by increased diastolic dimension and LV end-systolic volume when compared with the normal weight dogs. The present study also demonstrated that the obese dogs, both as a group and by breed (Chihuahua and Pomeranian), had decreased left ventricular free wall thickness, whereas LV mass and LV mass index were increased when compared to normal weight dogs. The same result was found in both Chihuahuas and in Pomeranian separately. Our study results are consistent with other studies of obese dog models [53,54,55,56]. However, in this study, LV mass and LV mass indices were found not to be related to left ventricular free wall thickness in the Pomeranian breed. This might have been be due to the small number of dogs of each breed.

The ECGs demonstrated obese dogs have an increased R-wave amplitude when compared to non-obese dogs, although this parameter was within normal limits in both groups. In addition, R wave amplitudes indicted ventricular depolarization. A possible mechanism for that depolarization might be eccentric hypertrophy, as indicated by the increased systolic-diastolic dimension, decreased left ventricular free wall thickness, and increased LV mass and LV mass index in obese dogs compared with non-obese dogs. Increased LV mass results in increased ventricular depolarization and increased amplitude of the R-wave. The present study thus suggests that a possible mechanism causing the decrease in cardiac systolic function might be intrinsic myocardial alterations resulting in increased cardiac mass which affect cardiac contractibility [53,54] without a specific breed-dependent effect in these small breed dogs.

HRV is also an indicator used to determine cardiac sympathovagal activity [13]. Although the normal range and the cut-off values of HRV in veterinary medicine have yet to be established, all dogs in this study evidenced good health. This study did show that HRV parameters were different in obese dogs when compared to non-obese dogs. In the time domain, we found that the mean NN, SDNN, SDANN, and ASDNN were statistically significantly lower in the obese dogs. In the frequency domain, the LF/HF ratio was higher in the obese dogs, indicating cardiac sympathovagal imbalance. Similarly, several studies in rats and humans have demonstrated that LF/HF ratios are statistically significantly higher in high-fat diet rats [57] and in obese humans [58]. In addition, a study in humans demonstrated that an imbalance of the sympathetic and parasympathetic mechanism creates a risk for cardiovascular diseases (CVDs) in overweight individuals [59]. The present study suggests that obesity could induce an increase in sympathetic activity and consequently induce HRV impairment.

In addition, a previous study showed that obese rats have increased oxidative stress which contributes to impaired cardiac autonomic balance [57]. Previous studies have also demonstrated that oxidative stress can lead to mitochondrial damage, causing damage to the electron transport system and upregulating the release of pro-apoptotic proteins, which then activate the caspase-cascade system, leading to apoptosis of neurons [60,61] and resulting in sympathovagal imbalance [62]. Thus, the relationship between MDA, HRV, and cardiac function could be due to the proposed mechanisms causing obesity-induced cardiac systolic dysfunction. Those mechanisms might be related to obesity-induced increases in oxidative stress MDA [30] and may lead to mitochondrial damage and cellular dysfunction [30,63], thus inducing HRV impairment and sympathovagal imbalance [62], resulting in a negative effect on cardiac systolic function [53,54]. Further studies need to be conducted to identify the specific mechanisms.

Additionally, breed-specific reference ranges for heart rate variability and MDA in dogs as well as cut-off values indicating impaired heart rate variability and/or systolic dysfunction remain to be established. Limitations of this exploratory study include the relatively small sample size, the evaluation of more than one breed, the lack of a pre-specified protocol, and the chance of selection bias. Further studies need to be conducted.

## 5. Conclusions

Obesity in dogs can induce an increase in oxidative stress, cardiac wall thickness, and HRV impairment and may also result in cardiac systolic dysfunction. Short-term HRV testing might have significant potential as a screening tool for cardiac autonomic dysfunction in obese dogs. Publicizing these relationships might also help build owner appreciation of the fact that appropriate weight control can retard autonomic function imbalance associated with obesity. Further breed-specific studies with larger sample sizes need to be conducted to confirm the hypotheses generated in this exploratory study.

## Figures and Tables

**Table 1:** Baseline characteristics of normal weight and obese groups. Mean ± SE (median, minimum–maximum) are reported.

Parameters	Normal Weight (*n* = 15)	Obese (*n* = 15)
Age	4.79 ± 0.5 (5, 2.0–8.0)	4.12 ± 0.33 (4.0, 1.8–8.0)
Body weight (Kg)	3.33 ± 0.41 (2.7, 1.6–7.2)	4.70 ± 0.33 * (4.8, 2.6–7.8)
Body condition score	4.44 ± 0.13 (4, 4.0–5.0)	7.20 ± 0.11 * (7, 7.0–8.0)
Systolic blood pressure (mmHg)	127 ± 4.55 (120, 106–165)	122 ± 6.14 (108, 97–166)
Diastolic blood pressure (mmHg)	77 ± 3.44 (59, 56–100)	68 ± 2.96 (61, 56–85)
Mean arterial blood pressure (mmHg)	100 ± 4.28 (80, 74–130)	90 ± 5.88 (75, 71–147)
Heart rate (beats/min)	129 ± 7.40 (120, 72–180)	128 ± 3.87 (122, 84–174)
Pulse rate (beats/min)	129 ± 7.40 (120, 72–180)	128 ± 3.87 (122, 84–174)
Respiratory rate (beats/min)	40 ± 4.41 (30, 24–80)	37 ± 3.80 (24, 24–52)
Rectal Temperature (°F)	101.7 ± 0.14 (101.2, 100.8–102.6)	101.8 ± 0.19 (101.4, 100.2–102.6)

* *p*-value < 0.05 vs. normal weight group.

**Table 2:** Blood profile in normal weight and obese groups. Mean ± SE (median, minimum–maximum) are reported.

Parameters	Normal Weight Group *(n* = 15)		Obese Group (*n* = 15)		Normal Range [46]
Packed cell volume (%)	45.40 ± 1.14	(46, 38–52)	49.89 ± 0.94 *	(49, 44–57)	35–57
Hemoglobin (g/dI)	14.88 ± 0.45	(14.8, 11.7–17.6)	16.76 ± 0.34	(16.6, 14.0–18.6)	11.9–18.1
RBC count (×10^6^ cells/µL)	6.13 ± 0.16	(6.01, 5.13–7.13)	6.97 ± 0.16 *	(6.9, 5.7–8.3)	4.95–7.87
MCV (fL)	73.37 ± 0.53	(73, 69.8–76.7)	72.68 ± 0.46	(72.8, 68.8–76.7)	60–77
MCHC (g/dI)	33.15 ± 0.25	(33.4, 30.4–34.3)	33.34 ± 0.3	(33.6, 31.3–35.5)	32.0–36.3
WBC count (×10^3^ cells/µL)	13.62 ± 0.95	(14.2, 6.43–17.8)	12.18 ± 0.96	(11.5, 8.2–22.2)	5–14.1
Segmented neutrophil (×10^3^ cells/µL)	9.29 ± 0.64	(10.12, 4.4–12.4)	7.74 ± 0.52	(7.49, 4.9–12.6)	2.9–12
Lymphocyte (×10^3^ cells/µL)	2.18 ± 0.12	(2.25, 0.37–4.45)	2.85 ± 0.56	(2.4, 1.2–10.2)	0.4–2.9
Monocyte (×10^3^ cells/µL)	1.07 ± 0.06	(1.17, 0.2–0.7)	0.83 ± 0.08	(0.91, 0.01–1.2)	0.1–1.4
Eosinophil (×10^3^ cells/µL)	0.80 ± 0.12	(0.92, 0.16–1.47)	0.59 ± 0.07	(0.6, 0.28–1.1)	0–1.3
Basophil (×10^3^ cells/µL)	0.04 ± 0.01	(0.03, 0–0.1)	0.04 ± 0.02	(0.02, 0.01–0.16)	0–0.14
Platelet count (×10^3^ cells/µL)	378.00 ± 27.9	(396, 231–560)	250.27 ± 31.5	(216, 66–452)	211–621
BUN (mg/dI)	17.41 ± 1.67	(17.85, 10.6–28.6)	18.25 ± 1.69	(17.4, 8.4–31.6)	8–28
Creatinine (mg/dI)	0.9 ± 4.55	(0.83, 0.6–1.22)	1.03 ± 0.07	(0.99, 0.71–1.83)	0.5–1.7
ALT (U/L)	50.27 ± 4.55	(47, 23–77)	82.71 ± 12.03	(76.5, 32–163)	10–109
ALP (U/L)	36.33 ± 4.17	(33, 18–69)	72.13 ± 15.11	(46, 23–204)	1–114
Total protein (g/dI)	7.65 ± 0.17	(7.6, 6.7–8.9)	7.43 ± 0.23	(7.3, 6.1–10)	5.4–7.5
Albumin (g/dI)	2.99 ± 0.10	(2.9, 2.5–3.7)	3.4 ± 0.08 *	(2.4, 2.7–3.8)	2.3–3.1
MDA (nmol/mL)	77.50 ± 4.23	(78.75, 42.5–115)	155.83 ± 12.8 *	(122.5, 95–307.5)	–

* *p*-value < 0.05 vs. normal weight group, *n* = 15 per group; RBC count, red blood cell count; MCV, mean corpuscular volume; MCHC, mean corpuscular hemoglobin concentration; WBC count, white blood cell count; BUN, blood urea nitrogen; ALT, alanine aminotransferase; ALP, alkaline phosphatase; MDA, malondialdehyde.

**Table 3:** Normalization with body weight of M-mode parameters in normal weight and obese groups. Mean ± SE (median, minimum–maximum) are reported.

M-Mode Parameters	Normalized Value				95% Prediction Interval (cm) [42]
	Normal Weight Group (*n* = 15)		Obese Group (*n* = 15)		
LVIDd (cm)	1.20 ± 0.09	(1.15, 0.69–1.63)	1.35 ± 0.05 *	(1.37, 1.03–1.47)	1.27–1.85
LVIDs (cm)	0.64 ± 0.04	(0.62, 0.42–0.83)	0.81 ± 0.03 *	(0.8, 0.52–0.96)	0.71–1.26
IVSd (cm)	0.46 ± 0.01	(0.46, 0.36–0.54)	0.46 ± 0.02	(0.45, 0.36–0.68)	0.29–0.59
IVSs (cm)	0.68 ± 0.02	(0.68, 0.56–0.85)	0.63 ± 0.02	(0.63, 0.49–0.75)	0.43–0.79
LVPWd (cm)	0.56 ± 0.02	(0.57, 0.41–0.72)	0.47 ± 0.02 *	(0.45, 0.36–0.58)	0.29–0.60
LVPWs (cm)	0.76 ± 0.02	(0.73, 0.61–0.84)	0.66 ± 0.02 *	(0.68, 0.54–0.77)	0.48–0.87
AO diameter (cm)	0.70 ± 0.02	(0.69, 0.72–1.5)	0.71 ± 0.01	(0.7, 0.62–0.78)	0.63–0.96
LA diameter (cm)	0.81 ± 0.03	(0.83, 0.63–1.04)	0.81 ± 0.03	(0.80, 0.6–1.0)	0.59–0.97

* *p*-value < 0.05 vs. normal weight group, *n* = 15 per group; LVIDd, left ventricular internal diameter end-diastole; LVIDs, left ventricular internal diameter end-systole; IVSd, interventricular septum thickness end-diastole; IVSs, interventricular septum thickness end-systole; LVPWd, left ventricular posterior wall end-diastole; LVPWs, left ventricular posterior wall end-systole; AO, aortic root; LA, left atrium.

**Table 4:** Cardiac function from M-mode parameters in normal weight and obese groups. Mean ± SE (median, minimum–maximum) are reported.

Echocardiographic Parameters	Normal Weight Group (*n* = 15)		Obese Group (*n* = 15)	
LA/AO	1.17 ± 0.03	(1.17, 0.91–1.38)	1.17 ± 0.05	(1.19, 0.82–1.54)
EDV (mL)	10.37 ± 2.2	(6.54, 1.78–35.44)	16.88 ± 0.94 *	(15.4, 8.22–25.7)
EF (%)	80.33 ± 1.84	(81.49, 61.4–90.5)	71.30 ± 1.51 *	(72.1, 60.4–82.55)
ESV (mL)	2.13 ± 0.49	(1.2, 0.32–6.68)	462 ± 0.58 *	(3.84, 1.45–11.0)
FS (%)	47.60 ± 1.82	(47.5, 29.33–57.6)	39.38 ± 1.43 *	(39.7, 30.8–48.3)
IVS% (%)	51.19 ± 4.41	(46.6, 20.5–80.28)	34.45 ± 4.38 *	(33.9, 11.4–73.7)
IVS/LVPW	0.90 ± 0.04	(0.89, 0.63–1.38)	1.08 ± 0.05	(0.93, 0.7–1.63)
LV mass (g)	18.57 ± 2.18	(15.9, 10.28–32.47)	28.04 ± 2.06 *	(26.3, 18.9–42.3)
LV mass index (g/m^2^)	84.64 ± 4.50	(78.7, 64.03–119.0)	97.45 ± 4.06 *	(94.8, 75.2–131.0)
LVPW% (%)	36.87 ± 3.54	(34.66, 18.6–74.12)	39.96 ± 5.18	(33.5, 12.4–77.8)

* *p*-value < 0.05 vs. normal weight group LA/AO, left atrial on aortic ratio; EDV, end-diastolic volume; %EF, %ejection fraction; ESV, end-systolic volume; %FS, %fractional shortening; IVS%, fractional thickening of the IVS; IVS/LVPW, ratio of interventricular septum thickness and left ventricular posterior wall thickness; LV mass, left ventricular mass; LV mass index, left ventricular mass index; LVPW%, percentage thickening of left ventricular posterior wall.

**Table 5:** Normalization with body weight of M-mode parameters compared between breed in normal weight and obese groups. Mean ± SE (median, minimum–maximum) are reported.

M-Mode Parameters	Normal Weight Group		Obese Group		95% Prediction Interval (cm) [42]
	Chihuahua (*n* = 9)	Pomeranian (*n* = 6)	Chihuahua (*n* = 8)	Pomeranian (*n* = 7)	
LVIDd (cm)	1.14 ± 0.06 (1.12, 0.69–1.36)	1.24 ± 0.12 (1.37, 0.94–1.5)	1.34 ± 0.03 * (1.35, 1.21–1.44)	1.35 ± 0.05 ^†^ (1.39, 1.03–1.47)	1.27–1.85
LVIDs (cm)	0.61 ± 0.04 (0.61, 0.46–0.82)	0.67 ± 0.07 (0.74, 0.42–0.83)	0.75 ± 0.03 * (0.72, 0.7–0.89)	0.86 ± 0.05 ^†^ (0.85, 0.8–0.96)	0.71–1.26
IVSd (cm)	0.46 ± 0.01 (0.46, 0.4–0.53)	0.46 ± 0.03 (0.46, 0.36–0.54)	0.45 ± 0.02 (0.45, 0.38–0.52)	0.48 ± 0.03 (0.46, 0.37–0.68)	0.29–0.59
IVSs (cm)	0.68 ± 0.02 (0.68, 0.6–0.75)	0.71 ± 0.05 (0.7, 0.56–0.85)	0.65 ± 0.02 (0.63, 0.56–0.72)	0.61 ± 0.04 (0.57, 0.49–0.75)	0.43–0.79
LVPWd (cm)	0.56 ± 0.03 (0.53, 0.44–0.72)	0.56 ± 0.04 (0.59, 0.41–0.64)	0.48 ± 0.02 * (0.45, 0.43–0.58)	0.46 ± 0.03 ^†^ (0.44, 0.36–0.57)	0.29–0.60
LVPWs (cm)	0.75 ± 0.02 (0.75, 0.67–0.84)	0.76 ± 0.02 (0.76, 0.71–0.81)	0.66 ± 0.03 * (0.65, 0.57–0.77)	0.63 ± 0.03 ^†^ (0.64, 0.54–0.76)	0.48–0.87
AO diameter (cm)	0.71 ± 0.03 (0.71, 0.59–0.88)	0.67 ± 0.02 (0.69, 0.58–0.74)	0.73 ± 0.02 (0.74, 0.64–0.78)	0.68 ± 0.01 (0.67, 0.62–0.74)	0.63–0.96
LA diameter (cm)	0.82 ± 0.04 (0.86, 0.63–1.04)	0.80 ± 0.04 (0.82, 0.69–0.84)	0.84 ± 0.03 (0.83, 0.73–0.94)	0.81 ± 0.05 (0.75, 0.7–1.0)	0.59–0.97

* *p*-value < 0.05 vs. Chihuahua normal weight group, ^†^ *p*-value < 0.05 vs. Pomeranian normal weight group. LVIDd, left ventricular internal diameter end-diastole; LVIDs, left ventricular internal diameter end-systole; IVSd, interventricular septum thickness end-diastole; IVSs, interventricular septum thickness end-systole; LVPWd, left ventricular posterior wall end-diastole; LVPWs, left ventricular posterior wall end-systole; AO, aortic root; LA, left atrium.

**Table 6:** Cardiac function from M-mode parameters by breed in normal weight and obese groups. Mean ± SE (median, minimum–maximum) are reported.

Echocardiographic Parameters	Normal Weight Group		Obese Group	
	Chihuahua (*n* = 9)	Pomeranian (*n* = 6)	Chihuahua (*n* = 8)	Pomeranian (*n* = 7)
LA/AO	1.16 ± 0.04 (1.16, 0.91–1.38)	1.19 ± 0.05 (1.21, 1.0–1.32)	1.17 ± 0.05 (1.21, 0.97–1.33)	1.17 ± 0.08 (1.14, 0.82–1.54)
EDV (mL)	8.10 ± 1.43 (6.54, 4.3–16.3)	11.8 ± 3.48 (13.2, 3.31–19.23)	14.24 ± 1.34 * (12.9, 12.11–22.13)	17.98 ± 1.89 (16.4, 14.7–25.7)
EF (%)	79.83 ± 2.73 (81.5, 61.43–90.45)	81.16 ± 2.14 (81.61, 72.7–87.2)	76.10 ± 2.73 (77.98, 61.18–82.5)	70.20 ± 2.44 ^†^ (69.5, 60.4–82.35)
ESV (mL)	1.56 ± 0.43 (1.1, 0.69–4.76)	3.28 ± 1.02 (3.26, 0.38–6.68)	3.36 ± 0.35 * (3.35, 2.17–5.07)	4.85 ± 0.87 (4.74, 1.45–10.1)
FS (%)	46.37 ± 2.69 (47.0, 29.33–57.65)	48.03 ± 2.12 (48.23, 39.7–53.62)	42.34 ± 2.69 (43.07, 31.28–49.33)	36.47 ± 1.54 ^†^ (37.1, 30.8–42.9)
IVS% (%)	45.95 ± 4.78 (44.13, 29.47–75.1)	56.70 ± 7.92 (57.39, 30.93–80.28)	41.32 ± 2.08 (43.5, 33.95–45.3)	28.85 ± 4.80 ^†^ (27.5, 11.4–47.8)
IVS/LVPW	0.88 ± 0.03 (0.89, 0.72–1.07)	0.92 ± 0.12 (0.86, 0.75–1.38)	0.92 ± 0.06 (0.91, 0.7–1.16)	1.10 ± 0.10 (1.13, 0.78–1.63)
LV mass (g)	15.90 ± 1.89 (14.35, 9.65–27.3)	21.88 ± 4.29 (27.35, 10.77–29.75)	26.73 ± 2.66 * (24.5, 20.25–40.88)	29.09 ± 3.16 (25.13, 19.75–42.3)
LV mass index (g/m^2^)	76.80 ± 4.27 (75.18, 59.6–96.8)	92.16 ± 9.21 (94.09, 64.03–124.0)	96.65 ± 3.83 * (96.7, 81.8–113.5)	99.26 ± 7.83 (97.47, 71.12–124.6)
LVPW% (%)	35.88 ± 3.71 (38.5, 18.6–53.5)	38.35 ± 7.36 (32.5, 24.43–74.12)	38.43 ± 8.52 (31.48, 12.38–72.03)	41.30 ± 6.75 (39.36, 22.74–77.8)

* *p*-value < 0.05 vs. Chihuahua normal weight group. ^†^ *p*-value < 0.05 vs. Pomeranian normal weight group. LA/AO, left atrial on aortic ratio; EDV, end-diastolic volume; %EF, %ejection Fraction; ESV, end-systolic volume; %FS, % fractional shortening; IVS%, fractional thickening of the IVS; IVS/LVPW, ratio of interventricular septum thickness and left ventricular posterior wall thickness; LV mass, left ventricular mass; LV mass index, left ventricular mass index; LVPW%, percentage thickening of left ventricular posterior wall.

**Table 7:** Time domain of heart rate variability in normal weight and obese groups. Mean ± SE (median, minimum-maximum) are reported.

Parameters	Normal Weight Group (*n* = 15)		Obese Group (*n* = 15)	
Mean NN	638.90 ± 45.24	(602.50, 491–968)	511.44 ± 33.90 *	(567.00, 302–615)
SDNN	167.20 ± 12.76	(165.00, 100–236)	93.11 ± 8.68 *	(92.00, 52–141)
SDANN	113.70 ± 18.71	(108.00, 45–255)	41.89 ± 8.68 *	(36.00, 12–97)
ASDNN	127.30 ± 9.92	(127.00, 77–196)	81.00 ± 6.41 *	(85.00, 44–106)
RMSSD	62.50 ± 5.93	(57.50, 35–105)	44.67 ± 4.93 *	(45.00, 20–66)
pNN50	39.05 ± 4.57	(37.05, 15.1–66.7)	25.40 ± 5.12	(24.4, 2.8–48.3)

* *p*-value < 0.05 vs. normal weight group. Mean NN, average NN intervals; SDNN, standard deviation of all NN intervals; SDANN, standard deviation of the averages of NN intervals in all 5 min segments of the entire recording; ASDNN, average standard deviation of all 5 min R-R intervals; RMSSD, square root of the mean of the sum of the squares of differences between adjacent NN intervals; pNN50, percentage of successive NN intervals > 50 ms.

**Table 8:** Frequency domain of heart rate variability in normal weight and obese groups. Mean ± SE (median, minimum-maximum) are reported.

Parameters	Normal Weight Group (*n* = 15)	Obese Group (*n* = 15)
VLF (ms^2^)	3512.93 ± 536.43 (3984.11, 1384.71–5546.59)	5206.53 ± 1162.33 (4495.12, 1922.03–14030.22)
LF (ms^2^)	2253.93 ± 352.19 (1820.26, 1079.48–3994.45)	2735.11 ± 382.74 (2532.76, 1537.51–5094.60)
HF (ms^2^)	1428.65 ± 203.51 (1578.19, 501.33–2314.43)	1081.14 ± 100.56 (1187.22, 569.50–1393.20)
LF/HF ratio	1.69 ± 0.18 (1.74, 0.65–2.51)	2.58 ± 0.31 * (2.44, 1.37–4.69)

* *p*-value < 0.05 vs. normal group. VLF, very low frequency; LF, low frequency; HF, high frequency; LF/HF ratio, low frequency/high frequency ratio.

**Table 9:** ECG parameters in normal weight and obese groups. Mean ± SE (median, minimum–maximum) are reported.

ECG Parameters	Normal Weight Group (*n* = 15)	Obese Group (*n* = 15)	Normal Range in Dogs [47]
Heart rate (bpm)	130 ± 6.58 (120, 80–180)	127 ± 5.87 (134.5, 100–160)	60 to170
P wave amplitude (mV)	0.20 ± 0.01 (0.2, 0.1–0.3)	0.22 ± 0.01 (0.2, 0.1–0.3)	<0.4
P wave duration (s)	0.04 ± 0.01 (0.04, 0.04–0.04)	0.04 ± 0.01 (0.04, 0.04–0.04)	<0.04
PR interval (s)	0.08 ± 0.01 (0.08, 0.06–0.1)	0.08 ± 0.01 (0.08, 0.06–0.1)	0.06 to 0.13
R amplitude (mV)	1.20 ± 0.09 (1.20, 0.7–1.8)	1.50 ± 0.09 * (1.4, 1.0–2.15)	>0.5 mV but <3 mV
QRS duration (s)	0.05 ± 0.01 (0.04, 0.04–0.08)	0.06 ± 0.01 (0.06, 0.04–0.08)	<0.07
ST segment (mV)	0.12 ± 0.02 (0.1, 0–0.2)	0.09 ± 0.03 (0.05, 0.02–0.25)	Elevation or depression > ±0.2 mV from baseline
T wave amplitude (mV)	0.20 ± 0.04 (0.15, 0.1–0.5)	0.27 ± 0.03 (0.25, 0.05–0.4)	< ±0.5 to 1 mV in all lead
QT wave interval (s)	0.18 ± 0.01 (0.2, 0.1–0.2)	0.18 ± 0.01 (0.18, 0.16–0.24)	0.15 to 0.24
Mean electrical axis (degrees)	−71.92 ± 5.84 (75, 30–105)	−58.60 ± 3.70 (59, 40–85)	From −45° to −147°

* *p*-value < 0.05 vs. normal weight group. All measurements were evaluated using lead II tracing at 25 mm/s paper speed and calibration 10 mm/mV. ECG, electrocardiogram; bpm, beat per minute.

## Abbreviations

ALP	Alanine aminotransferase	LVIDd	left ventricular internal diameter at end-diastole
ALT	Alkaline phosphatase	LVIDs	left ventricular internal diameter at end-systole
AO	Aortic root	LVPWd	Left ventricular posterior wall at end-diastole
ASDNN	Average standard deviation of all 5 min R-R intervals	LVPWs	Left ventricular posterior wall at end-systole
BCS	Body condition score	MCV	Mean corpuscular volume
BP	Blood pressure	MCHC	Mean corpuscular hemoglobin concentration
bpm	Beat per minute	MDA	Malondialdehyde
BUN	Blood urea nitrogen	MEA	Mean electrical axis
BW	Body weight	Mean NN	Average NN intervals
ECG	Electrocardiography	mV	Milli-volt
EF	Ejection fraction	pNN50	Percentage of successive NN intervals >50 ms
ESV	End-systolic volume	RBC	Red blood cell
FS	Fractional shortening	RMSSD	Root mean square of the sum of the squares of differences between adjacent NN intervals
HF	High frequency	ROS	Reactive oxygen species
HFnu	High frequency in normalized unit	SDANN	Standard deviation of the averages of NN intervals in 5 min
HRV	Heart rate variability	SDNN	Standard deviation of all NN intervals
IVSd	Interventricular septum thickness at end-diastole	SDRR	Standard deviation of all R-R intervals
IVSs	Interventricular septum thickness at end-systole	TP	Total power
LA	Left atrium	ULF	Ultra-low frequency
LF	Low frequency	VLF	Very low frequency
LFnu	Low frequency in normalized unit	VVTI	Vasovagal tonus index
LF/HF	Low frequency per high frequency	WBC	White blood cell
LV	Left ventricular		
